# Increasing ventilation reduces SARS-CoV-2 airborne transmission in schools: A retrospective cohort study in Italy's Marche region

**DOI:** 10.3389/fpubh.2022.1087087

**Published:** 2022-12-09

**Authors:** Giorgio Buonanno, Luca Ricolfi, Lidia Morawska, Luca Stabile

**Affiliations:** ^1^Department of Civil and Mechanical Engineering, University of Cassino and Southern Lazio, Cassino, Italy; ^2^International Laboratory for Air Quality and Health, Queensland University of Technology, Brisbane, QLD, Australia; ^3^Department of Psychology, University of Turin, Turin, Italy; ^4^David Hume Foundation, Turin, Italy

**Keywords:** ventilation, airborne transmission, school cohort study, Coronavirus disease, disease control strategies

## Abstract

**Introduction:**

While increasing the ventilation rate is an important measure to remove inhalable virus-laden respiratory particles and lower the risk of infection, direct validation in schools with population-based studies is far from definitive.

**Methods:**

We investigated the strength of association between ventilation and SARS-CoV-2 transmission reported among the students of Italy's Marche region in more than 10,000 classrooms, of which 316 were equipped with mechanical ventilation. We used ordinary and logistic regression models to explore the relative risk associated with the exposure of students in classrooms.

**Results and discussion:**

For classrooms equipped with mechanical ventilation systems, the relative risk of infection of students decreased at least by 74% compared with a classroom with only natural ventilation, reaching values of at least 80% for ventilation rates >10 L s^−1^ student^−1^. From the regression analysis we obtained a relative risk reduction in the range 12%15% for each additional unit of ventilation rate per person. The results also allowed to validate a recently developed predictive theoretical approach able to estimate the SARS-CoV-2 risk of infection of susceptible individuals via the airborne transmission route. We need mechanical ventilation systems to protect students in classrooms from airborne transmission; the protection is greater if ventilation rates higher than the rate needed to ensure indoor air quality (>10 L s^−1^ student^−1^) are adopted. The excellent agreement between the results from the retrospective cohort study and the outcome of the predictive theoretical approach makes it possible to assess the risk of airborne transmission for any indoor environment.

## Introduction

The acceleration of the research activity inspired by the COVID-19 pandemic revealed that airborne transmission is the main route of transmission for many respiratory infectious diseases with respect to other routes which were erroneously considered dominant in the twenteeth century (i.e., those not occurring *via* airborne route, such as contact) (1). Indeed, the prevalence of the airborne transmission amongst the different transmission routes was recognized by public health authorities much later even if a number of studies warned about the transmission routes of respiratory diseases at early stage of the pandemic (2). Only in December 2021, WHO updated one page in its website to clearly introduce the term “airborne transmission” [WHO Coronavirus disease (COVID-19): How is it transmitted? December 23, 2021. https://www.who.int/news-room/questions-and-answers/item/coronavirus-disease-covid-19-how-is-it-transmitted]. However, the description of the virus as “airborne” continues to be almost completely absent from public WHO communications and consequently from protective efficacy actions resulting, in fact, in the inability to control the pandemic. To date, few studies have examined the direct impact of ventilation on indoor transmission (3) but the SARS-CoV-1 outbreaks (4) in 2004, the MERS-CoV outbreaks (5) and the current SARS-CoV-2 pandemic (6, 7) have given a new impetus to research in this field, leading to new evidence and raising awareness of the importance of ventilation and indoor air quality for public health as well as clearly demonstrating the key role of an engineering approach to fighting airborne diseases (8). To this end, both mechanical ventilation systems, able to dilute the concentration of contaminants in the air with pathogen-free outdoor air, and air cleaners/purifiers, able to remove virus-laden respiratory particles from indoors thanks to different air filtration techniques, can be considered valuable solutions (9).

Schools represent a critical indoor environment due to the high crowding indexes (number of people relative to the size of the confined space), the long exposure times, and the possible inadequate clean (pathogen-free) air supply. In particular, some studies reported that schools do not amplify SARS-CoV-2 transmission, but rather reflect the level of transmission in the community (10–12). Nonetheless, several SARS-CoV-2 outbreaks in classrooms have been recognized worldwide (6, 13), and the situation has worsened with the Omicron variant, which is documented to spread amongst adolescents and children even faster than previous variants of concern (14, 15).

The objective of this retrospective cohort study was to investigate, through standardized methods for exposure assessment and statistical analysis, the strength of association between ventilation and SARS-CoV-2 airborne transmission in classrooms. To this end we exploited the data obtained from the government of Italy's Marche region which supported the installation of mechanical ventilation systems (MVSs) in approximately 3% of the schools in the region. The results obtained represent the very first proof of the effect of the ventilation against COVID-19 airborne transmission on a large-scale experiment.

## Methods

### Study design and participants

In March 2021, the government of central Italy's Marche region launched a 9 M€ call to fund the installation of MVSs in classrooms to prevent the airborne transmission of SARS-CoV-2 and limit the adoption of distance learning solutions. The funds enabled the installation of mechanical ventilation systems in 316 classrooms (in 56 schools applying for the funding). The population involved in this study consisted of 205 347 students at different educational stages (pre-school 14.6%, primary schools 33.1%, middle schools 18.9%, and high schools 33.4%) attending classes between 13 September 2021 and 31 January 2022. There were 1 419 schools in total included in the study, of which 56 were equipped with an MVS, for a total of 10 441 classrooms with an average occupancy of 20 students per classroom. A total of 10 125 classrooms relied on natural ventilation (i.e., ventilation due to leakage of the building and manual opening of the windows), while 316 were equipped with MVSs.

Infections were investigated in terms of clusters of cases that occurred rather than individual cases of infection; also in accordance with the Italian regulation that defined a cluster as the simultaneous presence in classrooms of 2 positive cases until December 2021 and 3 cases starting from January 2022 (16, 17). Temporal exposure was extrapolated from the regional weekly COVID-19 incidence and the relative risk reduction was correlated with the presence of the MVSs in the classrooms. The data was collected by the epidemiological observatory and by the school and infrastructure departments of the Marche region. The David Hume Foundation, a research institution specialized in data analysis, received from the Marche Region the data on the number of positive students in each class for 12 separate sub-periods from September 2021 to January 2022 to identify the clusters as defined above. The entire cohort is represented by the students in the classrooms equipped with MVSs: during the observation period, protective measures were adopted in Italian schools for students such as distancing, use of personal protective equipment (masks), and frequent opening of windows and doors to improve ventilation.

The MVSs installed in the classrooms are single room ventilation units, most of them equipped with heat recovery units and filters [F7 or G4 (18)] aimed at reducing the penetration of airborne particles from outdoor. The MVSs were switched on manually before classes start and they were run at a constant maximum flow rate all the school time long. No maintenance activities were performed in the limited period under investigation. The maximum air flow rates of the MVSs installed in the classrooms ranged from 100 to 1 000 m^3^ h^−1^ (with 25th, 50th, and 75th percentiles equal to 360 m^3^ h^−1^, 600 m^3^ h^−1^, and 800 m^3^ h^−1^, respectively) resulting in a ventilation rate per person (*Q*_*p*_) between 1.4 and 14 L s^−1^ student^−1^ for a classroom with an occupation density of 20 students and with a representative volume of 150 m^3^, as per the European survey (19). In case of natural ventilation AERs are typically < 0.5 h^−1^, i.e., < 1 L s^−1^ student^−1^, with reduced increases in mean ventilation in the case of occasional window openings (20). For the purposes of indoor air quality, an air change per hour (ACH) up to 5 h^−1^ is required in Italy (21), corresponding to a *Q*_*p*_ of 10 L s^−1^ student^−1^ for the above-mentioned occupation density and volume. The representative *Q*_*p*_ in European schools ranges from 1.5 to 9 L s^−1^ student^−1^, with lower rates being more representative for natural ventilation (22). Consequently, to stratify the analysis, we also introduced two sub-cohorts: (i) sub-cohort 1 represents classrooms with MVSs characterized by a *Q*_*p*_ between 1.4 and 10 L s^−1^ student^−1^ that meet the standard requirements of indoor air quality, also in relation to students' performance (23), and (ii) sub-cohort 2 includes classrooms with a *Q*_*p*_ > 10 L s^−1^ student^−1^ and up to 14 L s^−1^ student^−1^ and could represent health-based ventilation to protect from airborne transmission.

### Statistical analysis

We used simple descriptive statistics to characterize the study population, exposure, and risk reduction factors, summarizing quantitative data as means and categorical data as proportions.

Data on the number of positive students are provided as: (i) incidence cases (IC), i.e., the number of positive students counted only within clusters (provided separately for classrooms with and without MVSs and for different sub-periods); (ii) incidence proportions (IP), i.e., the number of positive students per 1 000 students (counted only within clusters and provided separately for classrooms with and without MVSs and for different sub-periods); and (iii) incidence proportion ratio (IPR), i.e., the ratio between the incidence proportion in classrooms with and without MVS.

The risk reduction factors considered in the statistical analysis are: i) the relative risk (RR), i.e., the outcome rate in the classrooms equipped with MVSs divided by outcome rate in the control group (i.e., classrooms without MVSs); ii) the relative risk reduction (RRR), defined as 1-RR, i.e., the proportional reduction of the events in the control group with respect to the investigated one (classrooms with MVS).

To assess the effect of the mechanical ventilation systems on risk reduction we adopted four indicators: i) the cardinal indicator y_1_ counts the total number of cases in each classroom, subtracts 1 (presumed primary case), and divides the result by the number of students; (ii) the cardinal indicator y_2_ which is similar to y_1_, except that for classrooms with 5 or more cases, only 4 secondary cases are always counted; (iii) the cardinal indicator y_3_ which is the arithmetic mean between y_1_ and y_2_; and (iv) the dummy indicator d_1_ which assumes a value of 1 if a cluster was identified in a classroom.

We developed several ordinary least squares and logistic regression models, including the confounding variables (educational stage and number of students per class) to estimate the net effect of the MVS. Details are reported in the Supplementary material. The data analysis was performed using IBM SPSS Statistics 28.0 and the results are presented as relative risks and 95% confidence intervals (CIs). We used the χ^2^ test and Fisher's exact test to compare proportions and the *F*-test and *t*-test for the statistical significance of the impact of the MVS.

### Role of the funding source

The government of the Marche Region as funder of the study had no role in the study design, data organization, data analysis, data interpretation, or writing of the report. LR and GB had full access to the data transmitted by the Marche Region and all the authors had final responsibility for the decision to submit for publication.

## Results

**Figure 1:**
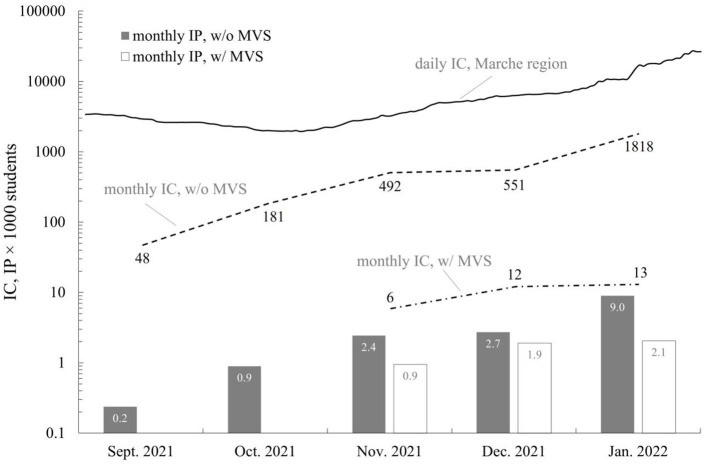
Daily cases in the Marche region, monthly incidence cases (IC) and incidence proportion (IP, per 1000 students) in classrooms with (w/) and without (w/o) mechanical ventilation systems (MVSs) from September 2021 to January 2022. For IC and IP the cases are counted only within clusters.

During the entire observation period we recorded 3 121 SARS-CoV-2 infected students within clusters (i.e., cases) in 1 004 classrooms, 31 in classrooms equipped with MVSs and 3 090 in classrooms without MVSs (Table 1). The monthly IP, expressed per 1 000 students, was not constant: from 13 September to 23 December 2021 it was lower than 7–31 January, as was the population of the Marche region (Figure 1). Indeed, the IPR for the entire period was equal to 0.32, but it was lower in the period of 7–31 January 2022 (IPR = 0.23) characterized by higher regional incidence cases (IC > 10 000 daily cases) than in the period of 13 September to 23 December 2021 (IPR = 0.45). The higher daily IC during the period 7–31 January 2022 is likely due to the Omicron variants spreading in Italy in that period which resulted more contagious than previous variants (24).

**Table 1:** Incidence cases (ICs), incidence proportions (IPs), and incidence proportion ratios (IPRs) observed in classrooms with and without mechanical ventilation systems (MVSs) during the periods of investigation.

**Parameter**	**Period of investigation**	**Classrooms without MVS**	**Classrooms with MVS**
Incidence cases, IC	13 September – 23 December 2021	1,272	18
	7–31 January 2022	1,818	13
	Entire period	3,090	31
Incidence proportion, IP (per 1000 students)	13 September – 23 December 2021	6.3	2.8
	7–31 January 2022	9.0	2.1
	Entire period	15.3	4.9
Incidence proportion ratio, IPR	13 September – 23 December 2021	0.45
	7–31 January 2022	0.23
	Entire period	0.32

For IC, IP and IPR the cases are counted only within clusters.

This result represents a key finding as it shows, for the very first time, the potentiality of a suitable mitigation strategy (i.e., ventilation) against airborne transmission based on a large and valuable data set.

The stratified analysis of the effect of the different ventilation rates (analysis by sub-cohorts) also demonstrated that higher ventilation rates provide greater RRRs (Table 2). Once again, when referring to the most conservative indicator (y_2_), the results showed that students in classrooms without mechanical ventilation had a 5-fold higher risk of infection compared with sub-cohort 2 (RR = 0.20), and a roughly 3-fold higher risk of infection compared with sub-cohort 1 (RR = 0. For each indicator, the classrooms equipped with MVSs were associated with reduced risk, indeed, even adopting the most conservative indicator (y_2_) a relative risk reduction of 74% was recognized29).

**Table 2:** Relative risks (RRs) and relative risk reductions (RRRs) for the four indicators for the different cohorts.

	**Entire cohort**	**Sub-cohort 1**	**Sub-cohort 2**
RR(y_1_)	0.19	0.21	0.15
RRR(y_1_)	0.81	0.79	0.85
RR(y_2_)	0.26	0.29	0.20
RRR(y_2_)	0.74	0.71	0.80
RR(y_3_)	0.23	0.25	0.17
RRR(y_3_)	0.78	0.75	0.83
RR(d_1_)	0.09	0.13	0.00
RRR(d_1_)	0.91	0.87	1.00

The association between ventilation and infection risk is significant regardless of the location, educational stages, and occupancy as clearly demonstrated in Table 3 where the classrooms were classified in 11 subsamples distinguished by provinces (four modalities), educational stages (four modalities), and number of students in the class (three modalities). The RRRs for the most conservative indicator (y_2_) are always positive (except in the case of Pesaro, dummy indicator, where −0.33 indicates an increase of the RR in respect to classrooms without MVSs): higher RRRs (even larger than 0.80) were detected in two provinces (Ancona and Macerata), in pre-schools (where no cases were detected, then resulting in null relative risk values), high schools, and in classrooms with more students.

**Table 3:** Relative risk reduction (RRR) ratios for four indicators and 11 subsamples.

		**RRR(y_1_)**	**RRR(y_2_)**	**RRR(y_3_)**	**RRR(d_1_)**
Province	Ancona	0.91	0.88	0.89	–
	Ascoli Piceno	0.67	0.52	0.60	–
	Macerata	0.88	0.84	0.86	–
	Pesaro	0.41	0.29	0.33	−0.33
Educational stage	Pre-schools	–	–	–	–
	Primary schools	0.80	0.71	0.76	–
	Middle schools	0.77	0.71	0.74	0.74
	High schools	0.84	0.80	0.82	–
Number of students in the classroom	Small classrooms	0.63	0.50	0.57	–
	Medium classrooms	0.83	0.77	0.80	0.83
	Large classrooms	0.85	0.78	0.81	–

RRRs are not provided in cases of null relative risk values.

## Discussion

The impact of SARS-CoV-2 on global public health, societies, and economies has been overwhelming. Various containment and mitigation strategies have been implemented by public health institutions to adequately contain the pandemic (25), but at the same time they did not convincingly support and propose to increase indoor ventilation to contain infections(26–29).

To the best of our knowledge, this is the first and largest retrospective cohort study in schools aimed at assessing the impact of mechanical ventilation in mitigating the risk of COVID-19 infection. The results demonstrate the effectiveness of the mechanical ventilation and the possibility of applying these techniques in a similar way in all indoor environments that represent the natural habitat of humans and which require complex, targeted management, not only of the control of thermal comfort, odors, perceived air quality, and energy use, but also of respiratory infections (30).

The outcomes of this retrospective cohort study demonstrate a lower incidence of COVID-19 cases in classrooms equipped with MVSs compared with classrooms with natural ventilation, with an IPR of 0.32 over the entire observation period and the entire cohort. The protection from contagion was even greater during the month of January 2022 (0.23), in the presence of high incidence at regional level (> 10,000 cases per day). This outcome suggests that the adoption of MVSs is even more noticeable and effective in periods (or with variants of concern) characterized by high virus circulation. This result was expected because of the key role ventilation plays in reducing occupational hazards according to the engineering level controls described in the traditional infection control hierarchy (31).

The incidence data allowed to estimate the positive impact of the mechanical ventilation on risk reduction: classrooms equipped with MVSs, in the most conservative case (indicator y_2_), reduces the likelihood of infection by 74%. A further interesting outcome of the paper is the evaluation of mechanical ventilation level on the RRR: in classrooms equipped with MVSs complying with Italian law in terms of indoor air quality (sub-cohort 1, *Q*_*p*_ up to 10 L s^−1^ student^−1^) the likelihood of infection for students is reduced, in the most conservative case, by 71% compared with a classroom relying only upon natural ventilation; whereas this reduction increases up to 80% in classrooms with MVSs providing a *Q*_*p*_ > 10 L s^−1^ student^−1^ (sub-cohort 2). It is therefore evident that pushing ventilation beyond 10 L s^−1^ student^−1^ (i.e., ACH > 5 h^−1^ for a classroom of 150 m^3^ with a density of 20 students) ensure a higher protection from respiratory infectious agents such as SARS-CoV-2. Thus, such ventilation rates >10 L s^−1^ student^−1^ could represent the future conditions of health-based protection to control not only thermal comfort, odors, perceived air quality, and energy use, but also respiratory infections.

We did find that the impact of the MVSs is greater than that estimated by the raw data (for all the indicators). As an example, if the regression models (details in the Supplementary material) obtained in the case of mechanical ventilation of 10 L s^−1^ student^−1^ are applied, and if we use the more conservative estimates, the average empirical RRR is 0.75, while the corrected value is 0.82 (corresponding to a RR of 0.18). This means that, once the confounding factors (educational stage and number of students per classroom) have been eliminated, the mechanical ventilation is even more incisive than it appeared from an empirical comparison between classes with and without mechanical ventilation.

The relative risk reduction for the entire cohort, evaluated as reported in the Supplementary material adopting the indicator y_2_ (chosen conservatively among the cardinal indicators), resulted in the range 15% (eq. S3) – 12% (eq. S5) for each additional unit of ventilation rate per person.

**Figure 2:**
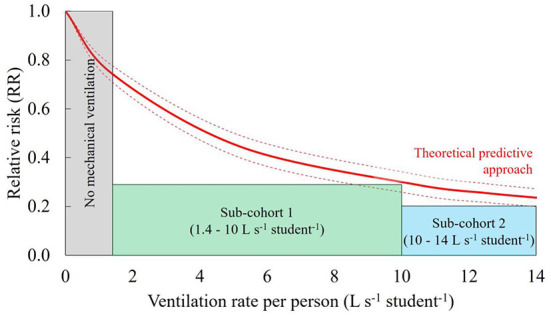
Comparison between the relative risk (RR) observed in the investigated classrooms and the RR estimated through the theoretical predictive approach for a specific scenario as a function of the ventilation rate per person, Qp.

The findings provided by the present retrospective cohort study are extremely important as they confirm the in-field effectiveness of ventilation in terms of risk reduction. Nonetheless, it would be desirable to provide prospective estimates of infection risk in different indoor environments. Recently, we developed a predictive theoretical approach that can estimate the SARS-CoV-2 risk of infection of susceptible individuals *via* the airborne transmission route when exposed to virus-laden particles emitted by an infected subject in an indoor environment (32–34). The risk of infection is estimated starting from the viral emission rate of the infected subject, the consequent viral concentration in the environment, the resulting viral dose of the exposed susceptible subject and, finally, the adoption of a proper dose–response model to allow the risk to be calculated. The novel aspect of the approach is the a priori evaluation of the viral emission of the infected subject on the basis of the viral load, the expiratory flow rate (influenced by the activity level), and the particle volume concentration expelled by the infectious person (affected by the expiratory activity, i.e., speaking, breathing, etc.). Major details of this predictive approach are reported in our previous papers and are not repeated here for the sake of brevity (32, 33, 35–37). Figure 2 shows the comparison between the RRs observed in the investigated classrooms and those estimated through the theoretical predictive approach for a specific scenario as a function of the ventilation rate per person. The scenario reported here considers: viral load and infectious dose typical of the Delta variant of concern [this variant was prevalent during the study period (35, 38, 39)], an average classroom volume of 150 m^3^, an infected student breathing only for the entire school time (5 h), exposed subjects performing only sitting/standing activities, and the effectiveness of masks on the reduction of the risk ranging from 0% (no mask) to 80% (actual reduction for respirators) (40). As expected, the simulated RR values decrease as a function of the ventilation rate per person with quite similar results to the observed RR: the RR obtained from the simulation at 14 L s^−1^ student^−1^ was equal to 0.24 ± 0.04.

The agreement between the results obtained from the retrospective cohort study and the values calculated through the predictive approach (32) is the second important finding of this paper. Indeed, this result represents a further validation of the approach through a retrospective cohort study that follows the experimental validation that we recently performed through an experimental study conducted under controlled conditions in a hospital room (41). Such validations confirm the possibility of extending the use of the approach, once the scenario has been defined, to any indoor environment of interest in addition to school classrooms and providing predictive estimates of the effectiveness of ventilation for different exposure scenarios and variants of concern.

To summarize, the findings of the present study could be extremely interesting for public authorities responsible for the safety of indoor environments, as well as for organizations and agencies working in the field of ventilation standards. In particular, public authorities should guarantee that highly crowded environments, like schools, are equipped with mechanical ventilation systems meeting the ventilation rate standards. This seems an obvious statement, but actually, most of the schools worldwide do not present a proper ventilation and mostly rely upon natural ventilation (19). Moreover, organizations such as the American Society of Heating, Refrigerating and Air-Conditioning Engineers and the Federation of European Heating, Ventilation and Air Conditioning Associations should improve their ventilation standards and explicitly consider infection control in addition to thermal comfort, odor control, perceived air quality, initial investment cost, energy use, and other performance issues in the management of indoor environments. Indeed, despite national regulations, the technical standards do not consider infection control when suggesting design air change per hour in indoor environments. As an example, the European standard EN-16798 (42, 43) suggests an *Q*_*p*_ in classrooms of > 10 L s^−1^ student^−1^ only for occupants with special needs (children, elderly, persons with disabilities, etc.; referred to as Category I) and for “non-low-polluted buildings” (i.e., buildings where no effort has been made to select low-emitting materials and where activities with emission of pollutants are not limited or prohibited).

Some limitations of this study should be noted. Firstly, further studies would provide deeper knowledge about the reduction of contagion risk in schools as a function of ventilation; for example, classrooms equipped with high ventilation should be investigated to identify a possible limit threshold beyond which the benefits in terms of risk reduction become negligible. Secondly, we considered a generalized operation of controlled mechanical ventilation systems at maximum flow rate. However, this was a reasonable hypothesis during the emergency period, which coincided with the observational period. Thirdly, our study was limited to SARS-CoV-2: other respiratory pathogens would require different ventilation rates per person.

## Data availability statement

The raw data supporting the conclusions of this article will be made available by the authors, without undue reservation.

## Author contributions

LR and GB conceived the study. LR, GB, LS, and LM contributed to the study design. LR contributed to the planning of statistical methods, data analysis, was involved in data collection, training of the study team, and data analysis. GB was the primary author of the manuscript. LM, LR, and LS contributed as senior authors on the manuscript draft and commented on the manuscript draft. All authors have seen and approved the submitted manuscript.
