# Supplementary material for: Increasing ventilation reduces SARS-CoV-2 airborne transmission in schools: A retrospective cohort study in Italy's Marche region
Source: Front Public Health. 2022 Dec 9;10:1087087. doi: 10.3389/fpubh.2022.1087087 (PMC9787545; doi:10.3389/fpubh.2022.1087087)
Supplement: Supplementary file 1 [file Data_Sheet_1.docx]

Supplementary Material

# Indicators

The indicators used, three cardinal (y_1_, y_2_, y_3_) and one dichotomous (d_1_), show positive and statistically significant correlations as reported in the correlation matrix of Table S 1 (sequence of eigenvalues: 2.732, 0.202, 0.066).

Table S 1. Correlation matrix amongst the selected indicators.

|  | y_1_ | y_2_ | y_3_ | d_1_ |
| --- | --- | --- | --- | --- |
| y_1_ | 1 | 0.91 | 0.98 | 0.80 |
| y_2_ |  | 1 | 0.97 | 0.89 |
| y_3_ |  |  | 1 | 0.86 |
| d_1_ |  |  |  | 1 |

The relationship between the mechanical ventilation system (MVS) and each of the four indicators was always statistically significant and such that the MVS always reduces the transmission of the virus. Table S 2 shows the results of the *t* test of equality of the means (for independent samples) for the three cardinal indicators (y_1_, y_2_, y_3_). Table S 3 summarizes the results of the significance tests for the impact of the MVS on the dummy d_1_.

Table S 2. Results of the *t* test of equality of the means for the cardinal indicators (y_1_, y_2_, y_3_).

| Indicator | *t* | *p-*value  (unilateral) |
| --- | --- | --- |
| y_1_ | 2.765 | 0.006 |
| y_2_ | 2.981 | 0.003 |
| y_3_ | 2.930 | 0.003 |

Table S 3. Results of the statistical tests for the dummy indicator (d_1_).

| Test | *p-*value  (bilateral) | *p-*value  (unilateral) |
| --- | --- | --- |
| Chi square | 0.001 | - |
| Fisher | <0.001 | <0.001 |
| Odds ratio (Confidence interval 95%) | 0.01–0.56 | |

In addition to mechanical ventilation, we have identified two additional factors (confounding parameters) that affect airborne transmission: the educational stage and the number of students per class. For these two factors, a dummy was introduced that distinguishes between compulsory school (CS, elementary and middle schools) and other educational stages, and a term of interaction between the number of students (NS) in the class and the absence of mechanical ventilation systems (1-MVS).

These two variables have been added to the mechanical ventilation to estimate the parameters of an ordinary regression model with a cardinal dependent variable (eq. S1) and of a logistic regression model with a dichotomous dependent variable (where y = 1 means that mechanical ventilation influences the airborne transmission, eq. S2).

$y=b_{0}+b_{1}\cdot ACH+b_{2}\cdot CS+b_{3}\cdot NS\cdot\left( 1-MVS \right)+error$ (S1)

$\frac{p\left( y=1 \right)}{1-p\left( y=1 \right)}=e^{\left( b_{0}+b_{1}\cdot ACH+b_{2}\cdot CS+b_{3}\cdot NS\cdot\left( 1-MVS \right) \right)}$ (S2)

The regressions were performed for all the indicators (3 cardinal, 1 dummy), obviously using the ordinary regression for the dependent variables y_1_, y_2_, y_3_, and the logistic regression for the dependent dummy variable d_1_. The effect of the MVS is always negative and statistically significant.

Table S 4. Main results of the regressions conducted for all four indicators (using the ordinary regression for the cardinal indicators y_1_, y_2_, y_3_ and the logistic regression for the dummy indicator d_1_) and the confounding parameters: b_1_ refers to mechanical air change per hour (ACH), b_2_ to compulsory schools (CS), and b_3_ to the number of students in the classroom (NS).

| Dependent  variable | Constant | | ACH (h^-1^) | | CS (-) | | NS (1-MVS) (-) | |
| --- | --- | --- | --- | --- | --- | --- | --- | --- |
|  | b_0_ | | b_1_ | | b_2_ | | b_3_ | |
|  | Coeff | Sig | Coeff | Sig | Coeff | Sig | Coeff | Sig |
| Ordinary regressions | | | | | | | | |
| y_1_ | 0.670 | 0.000 | -0.171 | 0.010 | 0.656 | 0.000 | 0.035 | 0.002 |
| y_2_ | 0.674 | 0.000 | -0.157 | 0.005 | 0.648 | 0.000 | 0.031 | 0.001 |
| y_3_ | 0.672 | 0.000 | -0.161 | 0.006 | 0.652 | 0.000 | 0.033 | 0.001 |
| Logistic regression | | | | | | | | |
| d_1_ | -3.535 | 0.000 | -0.625 | 0.014 | 0.655 | 0.000 | 0.080 | 0.000 |

# Relative risk estimate: direct approach

To quantify the effect of ventilation on airborne transmission, we compared the empirical relative risk (RR) per mechanical ventilation rate per person (*Q_p_*) with that obtained from a special logistic regression model. The mechanical *Q_p_* can be related to the air change per hour (ACH) of the classroom by considering a classroom occupation density of 20 students per classroom and a classroom volume of 150 m^3^.

Given two values of unit of mechanical *Q_p_* (*Q_p1_* and *Q_p2_*) and the corresponding estimated RR, we calculated the relative risk reduction (RRR) per unit *Q_p_*, (1-α), using the relationship:

$RR=\alpha^{\left( Q_{p2}-Q_{p1} \right)}$ ⇒ $\alpha={RR}^{\frac{1}{\left( Q_{p2}-Q_{p1} \right)}}$ (S3)

Applying this equation to the entire cohort we obtained α = 0.85, corresponding to an RRR for each additional unit of ventilation rate per person of 15%.

# RR estimate: logistic regression approach

The estimate of the RRR per unit ventilation rate per person can be also obtained by applying logistic regression to the conservative indicator y_2_ once it undergoes a dichotomization process. The dichotomization applied here is: the dummy y_2_ assumes the value of 1 with the same percentage of the observed cases. The α value can be obtained as follows:

$\alpha=e^{b_{1}}$ (S4)

The corresponding α value is 0.88, corresponding to an RRR for each additional unit of ventilation rate per person of 12%.

Table S 5. Main results of the logistic regressions conducted for the dummy variable y_2_ (after dichotomization) and the confounding parameters: b_1_ refers to mechanical air change per hour (ACH), b_2_ to compulsory schools (CS), and b_3_ to the number of students in the classroom (NS).

| Dependent  variable | Constant | | ACH (h^-1^) | | CS (-) | | NS (1-MVS) (-) | |
| --- | --- | --- | --- | --- | --- | --- | --- | --- |
|  | b_0_ | | b_1_ | | b_2_ | | b_3_ | |
|  | Coeff | Sig | Coeff | Sig | Coeff | Sig | Coeff | Sig |
| Logistic regression | | | | | | | | |
| Dummy (y_2_) | -3.276 | 0.000 | -0.133 | 0.011 | 0.746 | <0.001 | 0.033 | 0.002 |
